# Stress Intensity Factor of Semielliptical Surface Crack in Internally Pressurized Hollow Cylinder—A Comparison between BS 7910 and API 579/ASME FFS-1 Solutions

**DOI:** 10.3390/ma12071042

**Published:** 2019-03-29

**Authors:** Gabriel de C. Coêlho, Antonio A. Silva, Marco A. Santos, Antonio G. B. Lima, Neilor C. Santos

**Affiliations:** 1 Federal University of Campina Grande (UFCG), Campina Grande-PB 58429-140, Brazil; antonio.almeida@ufcg.edu.br (A.A.S.); santos.marco@ufcg.edu.br (M.A.S.); antonio.gilson@ufcg.edu.br (A.G.B.L.); 2 Federal Institute of Education, Science and Technology of Paraíba, João Pessoa-PB 58015-435, Brazil; neilorcesar@gmail.com

**Keywords:** stress intensity factor, semielliptical surface crack, hollow cylinder, finite element method, BS 7910, API 579/ASME FFS-1

## Abstract

The purpose of this research is to compare both British standard BS 7910 (2013) and American standard API 579/ASME FFS-1 (2016) stress intensity factor (SIF) solutions by considering a series of semielliptical surface cracks located in the external surface of a pressurized hollow cylinder in the axial direction. Finite element analysis was used as a comparison basis for both standards’ SIF results. The solution from the British standard provided consistent results compared to Finite Element (FE) results for crack depth not much higher than half the thickness in the deepest and surface-breaking points. Above those limits, the British standard’s solutions diverged quite a lot from the American standard, whose results followed FE values for every crack depth/thickness ratio tested with a maximum percentage difference of 1.83%.

## 1. Introduction

**Figure 1:**
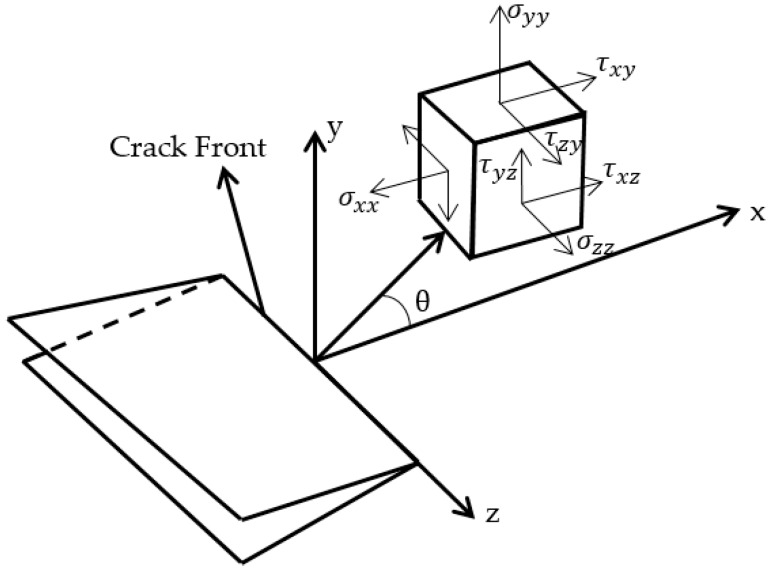
Stress state and polar coordinates system defined at crack front.

Stress intensity factor (SIF), first introduced by Irwin [1], is a physical quantity used as a control parameter to evaluate the critical state of a crack [2]. Assuming an isotropic material with linear elastic behavior, the stress field on any linear elastic body that holds a crack can be determined, according to Irwin [1], as follows:

(1) $$\sigma_{\mathrm{ij}}=\left(k/\sqrt{r}\right)f_{\mathrm{ij}}\left(\theta \right)+\overset{\infty }{\underset{m=0}{\sum }}A_{m}r^{m/2}g_{\mathrm{ij}}^{\left(m\right)}\left(\theta \right),$$

where $\sigma_{\mathrm{ij}}$ is the stress tensor; r and θ are the polar coordinates defined by the origin at the crack front, according to Figure 1; k is a constant; $f_{\mathrm{ij}}\left(\theta \right)$ are dimensionless functions; $A_{m}$ and $g_{\mathrm{ij}}^{\left(m\right)}\left(\theta \right)$ are higher-order terms for the amplitude and a dimensional function of θ for the m-th term, respectively.

Higher-order terms depend on the geometry, but the main term depends only on the $1/\sqrt{r}$ relation, and as r→0 the main term reaches an infinite value and the other terms in Equation (1) remain finite or null. Therefore, the stress near the crack front varies with $1/\sqrt{r}$ independently from the geometry of the body that holds the crack.

Regarding Equation (1), $f_{\mathrm{ij}}\left(\theta \right)$ and k depend on the load mode, but all three possible modes produce the $1/\sqrt{r}$ singularity. Therefore, replacing k by the SIF K such that $K=k\sqrt{2\pi }$ and omitting the higher-order terms, it is possible to obtain the elastic stress field around the crack front.

In practice, it is more reliable to calculate the SIF as a function of the remote stress acting on the cracked body, the crack geometry, and the geometry of the cracked body itself [2]. Considering a finite cracked body with remote stress inducing mode I, the SIF can be defined by

(2) $$K_{I}=Y\sigma \sqrt{\pi a},$$

where $K_{I}$ is the SIF in mode I loading; Y is a geometric parameter that takes into account the influence of geometry of the cracked body and the crack geometry itself; and $\underline{a}$ is a particular dimension of the crack.

When considering engineering critical assessment routes, such as the ones contained in the most popular industrial fitness-for-service standards API 579/ASME FFS-1 [3] and BS 7910 [4], estimation of $K_{I}$ might be performed considering each annex solution for a variety of crack and body geometries. Particularly, surface cracks in axial direction contained in cylindrical pressure vessels are commonly assessed for structural integrity and campaign extension.

**Figure 2:**
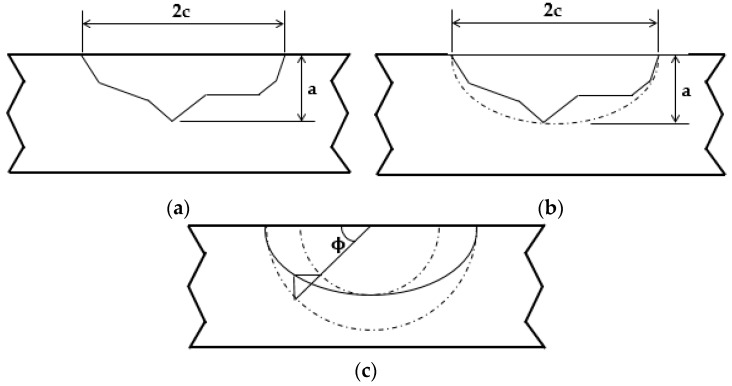
(**a**) Real crack with arbitrary dimensions; (**b**) idealized surface crack; (**c**) parametric semielliptical angular definition.

Real crack geometric configurations are arbitrary and do not exhibit standardized geometries. It would be very hard to define Y values considering each particular case. Therefore, it is common to idealize the crack geometry, conservatively considering only its maximum dimensions. This idealization is shown in Figure 2 for the surface crack.

The idealized surface crack dimensions are a and 2c, which represent the crack depth and crack width, respectively; and ϕ is defined as the parametric semielliptical angle.

The solution for the $K_{I}$ as applied for a surface crack under mode I, remote loading contained in a finite-thickness plate, can be determined as follows [5]:

(3) $$K_{I}=\sigma \sqrt{\frac{\pi a}{Q}}Y\left(a/c,\;a/t,\;c/W,\;\phi \right),0\leq \phi \leq \pi ,$$

where W is half the plate width; t is the plate thickness; and Q is defined as a crack geometry parameter that can be determined by the elliptical integral of a second kind, defined as follows [6]:

(4) Q2=∫0π2[1−c2−a2c2sen²(ϕ)]dϕ.

Equation (4) does not have a closed-form solution and a good approximation was introduced by Equation (5) with a maximum error of 0.13% when used. This equation is shown below:

(5) $$Q=\{\begin{array}{c} 1+1.464{\left(a/c\right)}^{1.65},\;a/c\leq 1\\ 1+1.464{\left(c/a\right)}^{1.65},\;a/c>1 \end{array}$$

The solution reported in Equation (5) was obtained after several finite element simulations were performed, such that a Y(a/c,a/t,c/W,ϕ) function could be derived.

The solution contained in Annex M of BS 7910 [4], as applied to the plate geometry with surface crack, is the same as proposed by Newman and Raju [5]. For the particular case of the surface crack contained in an internal pressurized hollow cylinder, the solution is simply a modification of the one presented by the same authors [5] considering a modification in the geometry parameters. This solution may also be seen in Coêlho [7].

The solution contained in Annex 9B of API 579/ASME FFS-1 [3] for the plate geometry is also the same as reported by [4]. However, the solution for the surface crack contained in an internally pressurized hollow cylinder is based on the weight function method, which allows for a way to infer the SIF for a non-uniform stress distribution [3], making it a very attractive method when solutions found in common engineering literature fail for a specific engineering application [8]. This solution may also be seen in Coêlho [7]. The American standard provides table values for the so-called influence coefficients for $0\leq t/R_{i}\leq 1$ and 0<a/t<0.8, where $R_{i}$ is the internal radius of the hollow cylinder.

Differences considering the SIF values obtained by both standards’ solutions have already been reported, by Sisan et al. [9] and Larrosa and Ainsworth [10], as leading to significant variations to the structural assessment. Although these studies have considered deep cracks, none of them considered crack depths outside the 0<a/t<0.8 range.

As more effort is placed on developing less conservative structural integrity assessment methodologies, knowing which solution provides less conservative SIF values is of great importance for industrial application of fitness-for-service standards. This study intends to compare both BS 7910 [4] and API 579/ASME FFS-1 [3] SIF solutions by considering a series of semielliptical surface cracks located in the external surface of an internally pressurized hollow cylinder in the axial direction.

## 2. Materials and Methods

For this study, a series of semielliptical surface cracks were considered. Crack dimensions are shown in Table 1, where t is the cylinder’s thickness. The a/c and $t/R_{i}$ ratios were fixed at 0.5 and 0.01667, respectively. For the purpose of simplicity for all analyses so that only a/t ratio will vary from within to outside the 0<a/t<0.8 range. The cracks were supposed to be located away from welding joints and heat affected zones from welding processes, neglecting any kind of residual or secondary stresses.

**Figure 3:**
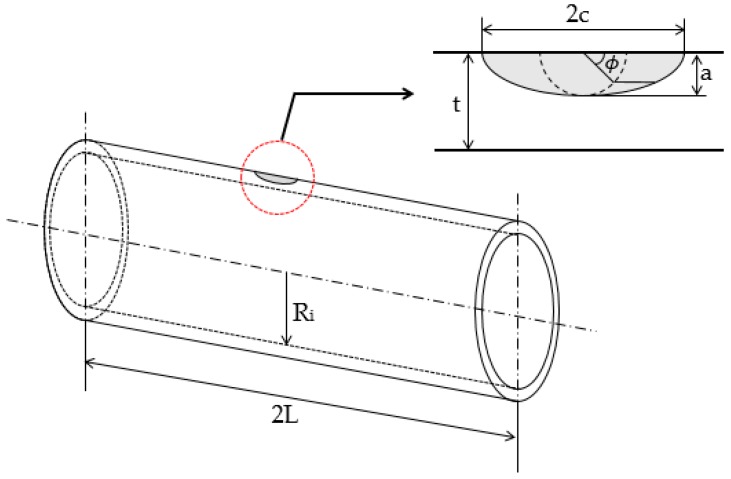
Hollow cylinder geometry with semielliptical surface crack at the external surface in the axial direction.

Hollow cylinder geometry and the crack location are shown in Figure 3 and cylinder dimensions are reported in Table 2. Internal pressure ($P_{\mathrm{int}}$) was set to 3.5 MPa and the material considered is a structural steel with Poisson’s ratio (ν) 0.3 and Young’s modulus (E) 204.5 GPa. The hollow cylinder is considered to have no dynamic loadings.

Primary membrane ($P_{m}$) and bending ($P_{b})$ stresses were calculated according to API 579/ASME FFS-1 [3], such that

(6) $$P_{m}=\frac{P_{\mathrm{int}}R_{i}}{t},$$

(7) $$P_{b}=\frac{-P_{\mathrm{int}}}{2}.$$

For the purpose of comparison between the solutions given by API 579/ASME FFS-1 [3] and BS 7910 [4] for the case explored here, finite element method (FEM) was used with ABAQUS^®^ commercial software (version 6.12, Dassault Systèmes^®^, Paris, France). The solutions and the numerical results were compared using the mean percentage difference, $\bar{d}$, such that

(8) $$\bar{d}=\frac{{\sum_{i=1}^{N}\left(\frac{\left|K_{I}^{A}-K_{I}^{\mathrm{MEF}}\right|}{K_{I}^{A}}\right)}_{i}}{N}\times 100,$$

where $K_{I}^{A}$ is the mode I loading SIF calculated by solutions; $K_{I}^{\mathrm{MEF}}$ is the mode I loading calculated by FEM; and N. is the number of SIF values extracted from the semielliptical geometry that corresponds to a specific ϕ value. Analogously, the percentage difference for a specific ϕ value, d, is defined as

(9) $$d=\frac{\left|K_{I}^{A}-K_{I}^{\mathrm{MEF}}\right|}{K_{I}^{A}}\times 100.$$

The hollow cylinder considered in Table 2 was modeled in ABAQUS^®^ commercial software, but the cylinder length could be reduced for the purpose of computational time reduction since, according to [12], a L/c≥10 ratio should be enough to neglect the length effect of the SIF. Therefore, for the FEM models, 2L was set to 800 mm.

Further, for the purpose of computational time reduction, a tie-constraint technique was used to decrease mesh density. This technique ties two surfaces together for the duration of the simulation, restraining each one of the slave surface nodes to have the same movement as the closest master surface nodes [13]. This technique was successfully used by Tipple and Thorwald [14] in the modeling of a corner crack in the neck–manhole junction of the pressure vessel and its hull for the estimation of SIF and crack propagation. Also, it has been successfully used by Coêlho et al. [15] for studying the capability of XFEM (eXtended Finite Element Method) to estimate SIF for surface cracks located in internally pressurized hollow cylinders compared to conventional FEM.

### Developed Finite Element (FE) Model

**Figure 4:**
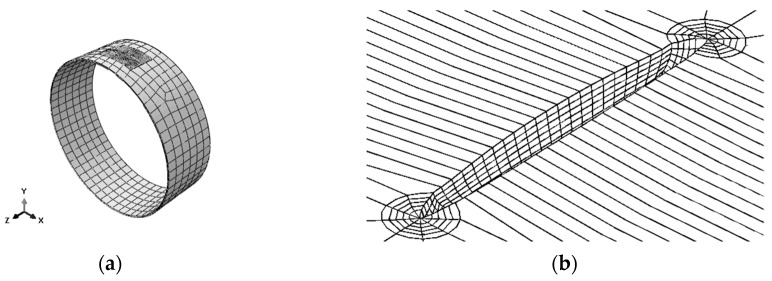
(**a**) Model used for finite element (FE) simulations of all cracks; (**b**) crack opening due to internal pressure loading.

Figure 4 illustrates the developed base model for the FE simulations. The only change in each model developed for every crack described in Table 1 is related to the variation of the crack dimensions in each geometric model. Figure 4a shows a high-density mesh, known as the slave instance, and a low-density mesh, known as the master instance; both instances are fixed to each other by the tie constraint technique. Figure 4b gives an example of a crack opening because of the internal pressure loading.

**Figure 5:**
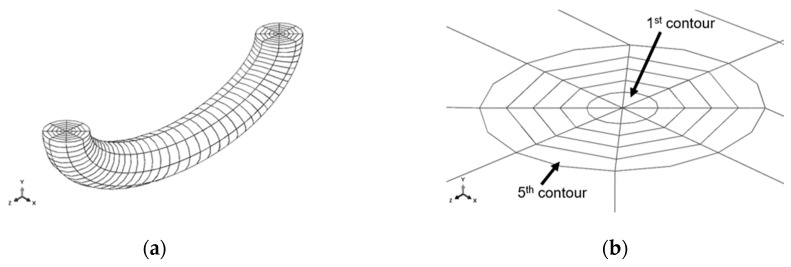
(**a**) Mesh details around crack front; (**b**) mesh contours around crack front.

C3D20R elements (hexahedral second-order elements with 20 nodes and reduced integration) were used in the master instance and in most part of the slave instance, with the exception of the first contour around the crack front, in which C3D15 elements (prismatic second-order elements with 15 nodes) were used. Mesh details around the crack front are shown in Figure 5, where it is possible to see the first contour using C3D15 elements and the other contours using C3D20R elements. Table 3 reports the values for each model developed here.

Regarding computational methodology, the models developed make use of ABAQUS^®^ explicit integration algorithm with contour integral method to explicit the stress intensity factor values and interaction integral for separating these values into the three loading modes.

## 3. Results

### FE Results

A model was developed for each crack and information regarding each numerical model that converged can be seen in Table 3. Table 4 summarizes the mean percentage difference for four slave instance global mesh sizes, h, regarding the solutions for SIF calculation being compared here. Convergence is set considering the mesh size that most approximated for one of the solutions. The highlighted $\bar{d}$ percentage values indicate which mesh converged for each of the crack models.

Figure 6, Figure 7, Figure 8, Figure 9, Figure 10, Figure 11, Figure 12, Figure 13, Figure 14, Figure 15, Figure 16 and Figure 17 show the mode I SIF profiles obtained for each crack modeled in this research. The profiles obtained are a function of the parametric angle ϕ, and both British and American standards solution results are shown for each crack in comparison to each profile obtained by FE simulations with the mesh sizes mentioned in Table 4.

## 4. Discussion

### 4.1. FE Result Analysis

**Figure 6:**
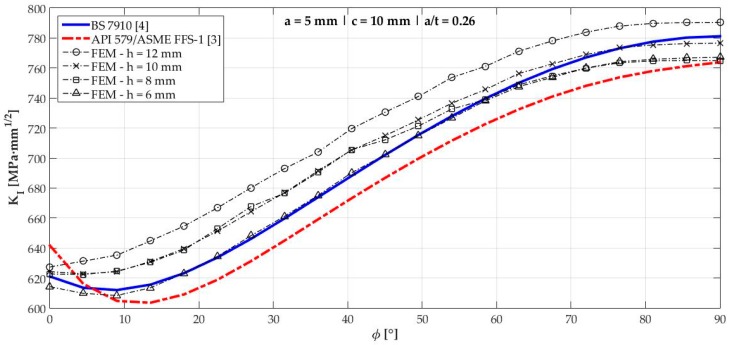
Mode I Stress intensity factor (SIF) profiles for crack 1 (a=5 mm, c=10 mm, and a/t=0.26).

**Figure 7:**
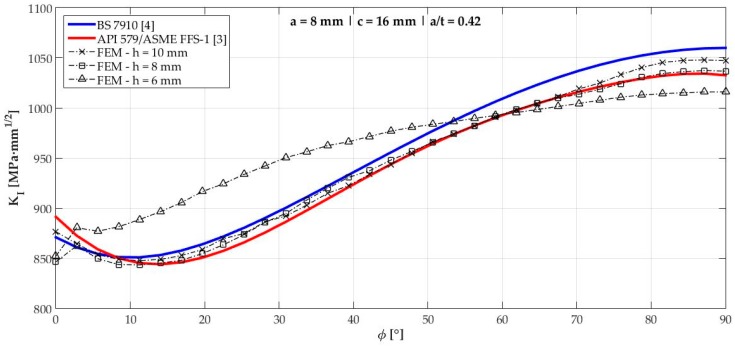
Mode SIF profiles for crack 2 (a=8 mm, c=16 mm, and a/t=0.42).

**Figure 8:**
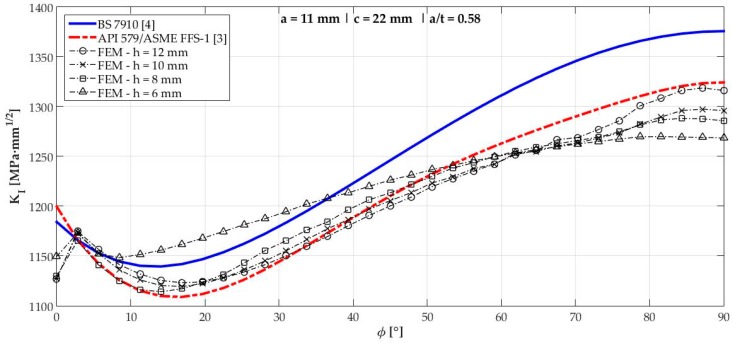
Mode I SIF profiles for crack 3 (a=11 mm, c=22 mm, and a/t=0.58).

**Figure 9:**
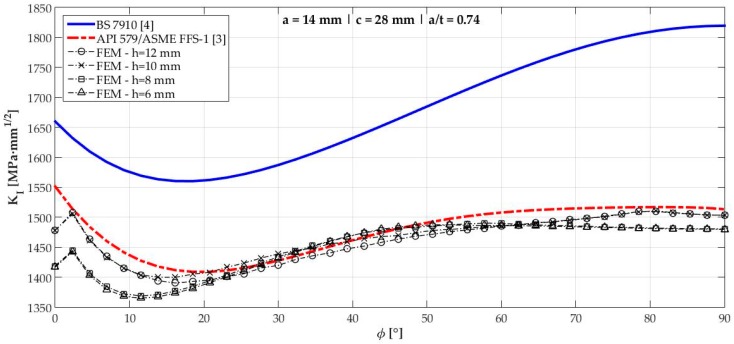
Mode I SIF profiles for crack 4 (a=14 mm, c=28 mm, and a/t=0.74).

**Figure 10:**
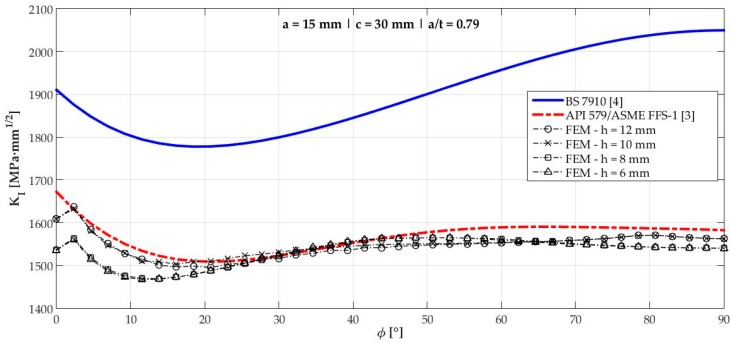
Mode I SIF profiles for crack 5 (a=15 mm, c=30 mm, and a/t=0.79).

**Figure 11:**
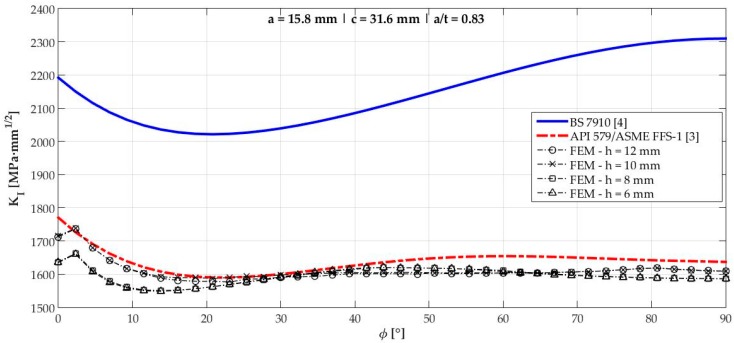
Mode I SIF profiles for crack 6 (a=15.8 mm, c=31.6 mm, and a/t=0.83).

**Figure 12:**
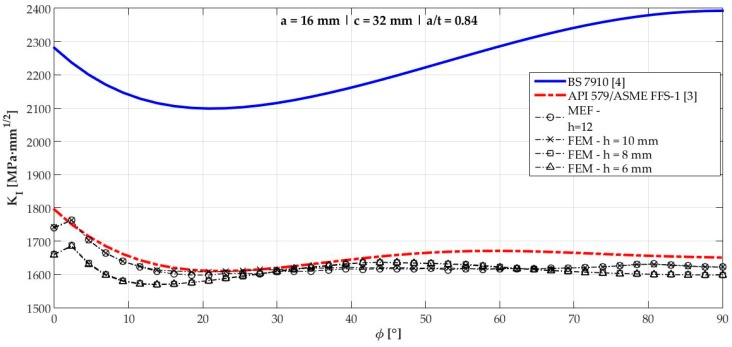
Mode I SIF profiles for crack 7 (a=16 mm, c=32 mm, and a/t=0.84).

**Figure 13:**
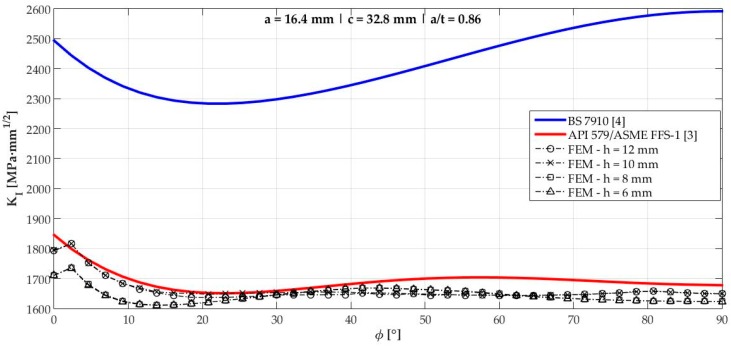
Mode I SIF profiles for crack 8 (a=16.4 mm, c=32.8mm, and a/t=0.86).

**Figure 14:**
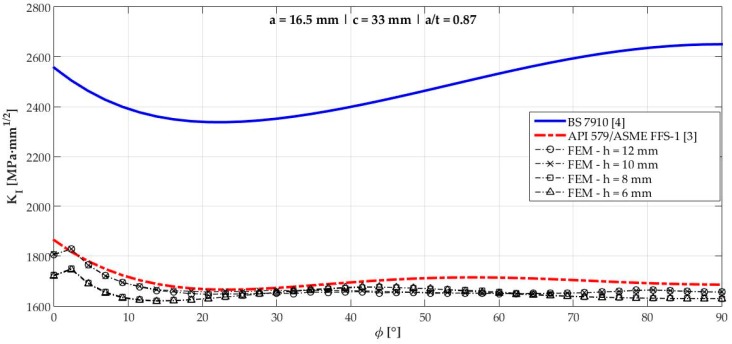
Mode I SIF profiles for crack 9 (a=16.5 mm, c=33 mm, and a/t=0.87.).

**Figure 15:**
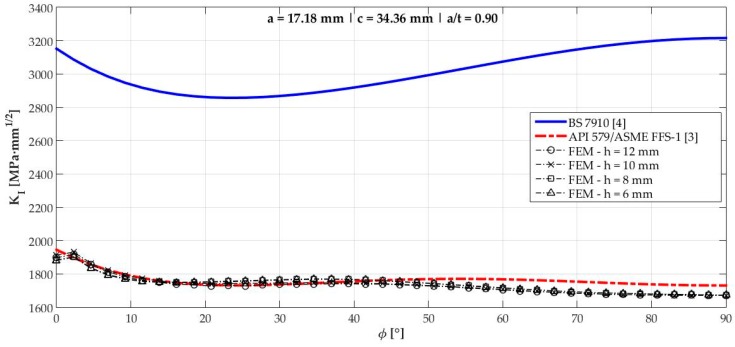
Mode I SIF profiles for crack 10 (a=17.18 mm, c=34.36 mm, and a/t=0.90).

**Figure 16:**
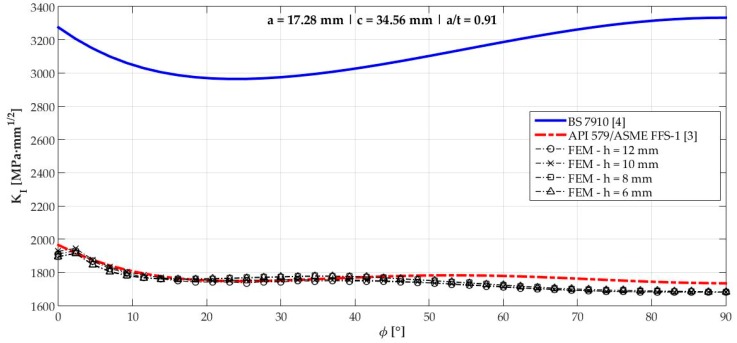
Mode I SIF profiles for crack 11 (a=17.28 mm, c=34.56 mm, and a/t=0.91).

**Figure 17:**
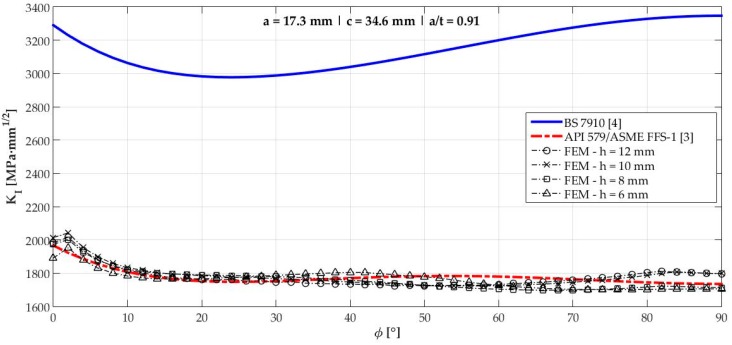
Mode I SIF profiles for crack 12 (a=17.3 mm, c=34.6 mm, and a/t=0.91).

From the analysis of Figure 6, Figure 7, Figure 8, Figure 9, Figure 10, Figure 11, Figure 12, Figure 13, Figure 14, Figure 15, Figure 16 and Figure 17, variations can be noticed in the SIF profile when the crack breaks the free surface [16], that is, when ϕ=0°, especially for deeper cracks. This behavior occurs because for materials whose Poisson’s ratio lies in the range of real and practical use values, it is quite hard to derive a precise SIF value when ϕ=0° [17], requiring that ν→0 for the $r^{-1/2}$ singularity to exist. For a deeper mathematical analysis, we recommend the work of Benthem [18].

Therefore, it is remarkable that the extracted SIF values when ϕ=0° cannot be trusted for structural integrity assessment. For this parametric semielliptical angular position, the closest FE extracted value was considered following at least one of the solutions.

### 4.2. Discussion of SIF Solutions

Table 5 summarizes the SIF values obtained considering the cracks analyzed here for British and American standards solutions, and the respective percentage differences, d, regarding FEM results.

The mode I SIF magnitudes when ϕ=0° and ϕ=90° were normalized by $\left(P_{m}+P_{b}\right)\sqrt{\left(\pi t\right)}$ magnitude for each BS 7910, API 579/ASME FFS-1, and FE results, resulting in $K_{I}^{\mathrm{norm}}$ such that

(10) $$K_{I}^{\mathrm{norm}}=\frac{K_{I}}{\left(P_{m}{+P}_{b}\right)\sqrt{\left(\pi t\right)}}.$$

**Figure 18:**
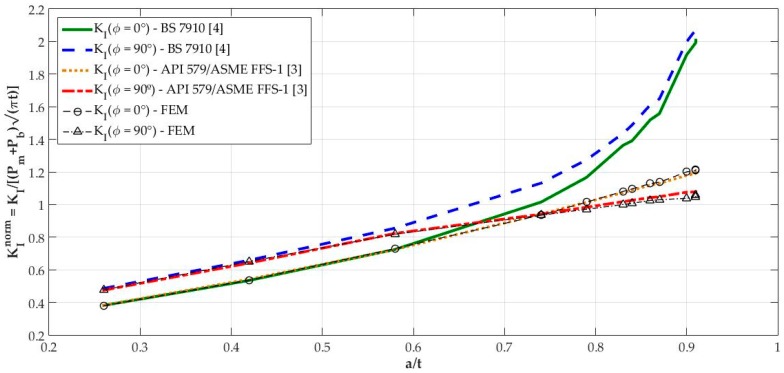
Normalized mode I SIF values versus a/t ratio.

Figure 18 shows the variation of $K_{I}^{\mathrm{norm}}$ with a/t ratio. It is clear that the solution from BS 7910 [4] provides consistent results compared to FE results for a/t<0.6 in ϕ=90° and a/t<0.7 when ϕ=0°. Above those limits, these solutions diverge quite a lot from API 579/ASME FFS-1 [3], whose results follow FE results for every a/t ratio tested with a maximum percentage difference of 1.83% for crack 10.

Such inconsistent results out of the limits mentioned are related to the origin of BS 7910 solution which, as already mentioned, is the same solution as a centered surface crack on a remote-tensioned finite plate with geometrical modifications. Its efficiency, however, had already been tested considering previous research of the same authors [19]. Raju and Newman [19] tested several a/t crack configurations in the 0.2<a/t<0.8 interval in comparison to other proposed solutions at the time. Considering $K_{I}$ when ϕ=90°, an error of ±5% was obtained until a/t<0.6, which is consistent with the obtained results shown in Table 5, where crack 3 (a/t=0.58) displayed a 4.33% difference from the FE result when ϕ=90°.

The capability of the API 579/ASME FFS-1 [3] solution to provide consistent results, when compared to FE results, is due to it being based on the weight function method, whose advantages were previously mentioned in the introduction of this paper. The standard API 579/ASME FFS-1 [3] only provides weighting parameters for the calculation of influence coefficients when a/t=0.0, 0.2, 0.4, 0.6, and 0.8. However, the use of linear interpolation for a/t ratios different from the ones provided within 0.0≤a/t≤0.8, and the use of linear extrapolation for a/t ratios outside of the upper limit of the mentioned range, were shown to be efficient for estimating these weighting parameters, even considering a light deviation from FE results for a/t>0.8.

### 4.3. Influence of Both Solutions on Structural Integrity Assessment

For the intent of exemplification of the influence of both solutions in structural integrity assessment, the results presented in Figure 18 were used to assess the hollow cylinder’s integrity in the framework of both American and British standards, engineering critical assessment routes. Herein, the analysis considers the Option 2 assessment route of BS 7910 [4] and Level 2 assessment route of API 579/ASME FFS-1 [3], whose methodologies consist of the use of the failure assessment diagram (FAD). For more details about the assessment routes, it is advised to consult each standard or check the work of Coêlho [7] for a consistent revision of these FAD methodology issues. A brief explanation of the FAD methodology is given below.

**Figure 19:**
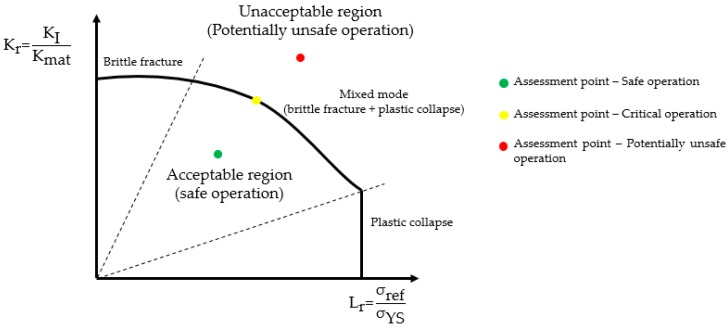
Schematic on the usage of failure assessment diagram (FAD) for structural integrity assessment.

Figure 19 shows a schematic of the usage of FAD methodology. The abscissa ($L_{r}$) is called the load ratio, which accounts for the load acting on the structure in the framework of the reference stress ($\sigma_{\mathrm{ref}}$, calculated considering body and crack geometries) and the material yield strength ($\sigma_{\mathrm{YS}}$). The ordinate ($K_{r}$) is called the toughness ratio, which accounts for the maximum SIF of the crack for a given load and the material fracture toughness ($K_{\mathrm{mat}}$). The FAD line is a such that $K_{r}=f\left(L_{r}\right)$, which may vary for each standard and the assessment level used (as the assessment level grows, a more refined and less conservative FAD is generated). The assessment point ($L_{r}$, $K_{r}$) is plotted on the FAD and if it lies under the FAD curve, the structure containing the crack is considered safe for operation. On the other hand, if the assessment point lies above the FAD curve, the structure is considered potentially unsafe for operation, and rerate is recommended before discarding. However, if the assessment point relies on the FAD curve, the crack is considered critical. The dashed lines show the failure regions predicted by the FAD, although these regions are only highlighted here for didactic purposes.

The Option 2 assessment route of BS 7910 [4] uses the following FAD curve:

(11) $$K_{r}={\left(\frac{{E\varepsilon }_{\mathrm{ref}}}{L_{r}\sigma_{Y}}+\frac{L_{r}^{3}\sigma_{\mathrm{YS}}}{2{E\varepsilon }_{\mathrm{ref}}}\right)}^{-1/2},$$

where $\varepsilon_{\mathrm{ref}}$ is the reference deformation corresponding to $\sigma_{\mathrm{ref}}$ at the stress–strain curve of the material, which will be estimated by the Ramberg–Osgood relation [20]. The material yield strength is 364 MPa, and the lower-bond fracture toughness was estimated as 3340.63 MPa·mm^1/2^ [7].

The Level 2 assessment route of API 579/ASME FFS-1 [3] uses the following FAD curve:

(12) $$K_{r}=\left[1-0.14{\left(L_{r}\right)}^{2}\right]\left\{0.3+0.7\exp\left[-0.65{\left(L_{r}\right)}^{6}\right]\right\}.$$

**Figure 20:**
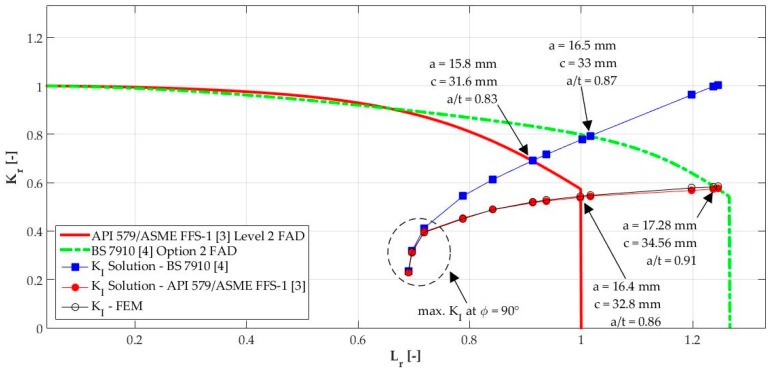
API 579/ASME FFS-1 [3] Level 2 and BS 7910 [4] Option 2 FAD assessment.

Figure 20 shows the application of both FADs considering the solutions provided by the American and British standards, as well as the results obtained by FE analysis. Both solutions evaluate, almost equally, the structural integrity of the cracked hollow cylinder at the first three assessment points with a maximum $K_{I}$ at ϕ=90° (a/t<6 at Figure 18). Subsequently, the change of the parametric location, from 90° to 0°, of the maximum $K_{I}$ is seen in Figure 20 considering the API 579/ASME FFS-1 [3] solution, which is in agreement with the FE results. This deviation from the solution provided by BS 7910 [4], whose maximum $K_{I}$ parametric location is 90° for all crack dimensions, provides an inaccurate estimation of the critical crack size. Although the FAD curves are different, considering only one FAD, the critical crack size provided by the American standard solution (and followed by the FE results) are higher than the ones provided by the British standard solution.

## 5. Conclusions

In this work, the comparison between mode I SIF solutions provided by worldwide standards, BS 7910 [4] and API 579/ASME FFS-1 [3], was performed considering an external semielliptical surface crack in an axial direction of an internally pressurized hollow cylinder.

The API 579/ASME FFS-1 [3] solution displayed excellent capability regarding the estimation of mode I SIF profile for every crack modeled here. The FE results showed little difference from this solution, even outside the 0<a/t<0.8 range, especially for the parametric semielliptical angular positions ϕ=0° and ϕ=90°, whose importance is quite great for structural integrity assessments.

BS 7910 [4] solution displayed poor results for mode I SIF for a/t>0.6 when ϕ=90°, and a/t>0.7 when ϕ=0°, in comparison to the FE results. The reason for that is attributed to the origin of this solution: A remotely tensioned plate with centered surface crack and geometric modifications.

## Figures and Tables

**Table 1:** Variation of crack dimensions.

Crack	a (mm)	c (mm)	a/t
1	5.00	10.00	0.26
2	8.00	16.00	0.42
3	11.00	22.00	0.58
4	14.00	28.00	0.74
5	15.00	30.00	0.79
6	15.80	31.60	0.83
7	16.00	32.00	0.84
8	16.40	32.80	0.86
9	16.50	33.00	0.87
10	17.18	34.36	0.90
11	17.28	34.56	0.91
12	17.30	34.60	0.91

**Table 2:** Geometric parameters for hollow cylinder. Data taken from Ramos [11].

Parameter	Value (mm)
Cylinder length (2L)	6000.00
Internal radius ($R_{i}$)	1139.75
Thickness (t)	19.00

**Table 3:** Information regarding each numerical model that converged.

Crack	Element Type	Number of Elements	Number of Nodes
1	C3D15 (1° contour) + C3D20R (remaining)	115,802	499,828
2		29,636	135,922
3		25,524	116,698
4		38,892	174,860
5		38,892	174,860
6		38,892	174,860
7		38,892	174,860
8		38,892	174,860
9		38,892	174,860
10		35,754	162,028
11		35,754	162,028
12		239,820	1,008,164

**Table 4:** Convergence of mesh size for each model.

Crack	a/t	Mean Difference Percentage, $\bar{d}$										
		h = 12 mm		h = 10 mm		h = 8 mm		h = 6 mm	
		BS	API	BS	API	BS	API	BS	API
1	0.26	3.32%	5.49%	1.46%	3.51%	1.63%	2.99%	0.59%	1.91%
2 *	0.42	-	-	1.30%	0.60%	3.48%	3.45%	3.42%	3.51%
3	0.58	3.49%	1.11%	3.69%	1.31%	3.53%	1.31%	3.31%	2.69%
4	0.74	12.88%	1.15%	12.55%	0.98%	13.39%	1.92%	13.49%	2.01%
5	0.79	18.56%	1.35%	18.36%	1.24%	19.18%	2.11%	19.20%	2.18%
6	0.83	25.07%	1.77%	24.92%	1.59%	25.72%	2.59%	25.74%	2.62%
7	0.84	27.00%	1.85%	26.87%	1.68%	27.65%	2.68%	27.67%	2.71%
8	0.86	31.38%	1.97%	31.25%	1.79%	32.00%	2.81%	32.02%	2.84%
9	0.87	32.58%	2.19%	32.47%	2.02%	33.20%	3.05%	33.24%	3.11%
10	0.90	42.60%	1.98%	42.42%	1.94%	42.21%	1.98%	42.27%	1.98%
11	0.91	44.37%	2.14%	44.20%	1.95%	44.00%	1.96%	44.06%	1.97%
12	0.91	43.43%	1.89%	43.22%	2.14%	43.88%	2.18%	43.83%	1.86%

* It was not possible to distribute prismatic second-order elements with mesh size of h = 12 mm along the semielliptical crack front for crack 2 with no element distortion error.

**Table 5:** Maximum mode I SIF values obtained from BS 7910 [4] and API 579/ASME FFS-1 [3] solutions and finite element method (FEM) results.

Crack	FEM		BS 7910 [4]			API 579/ASME FFS-1 [3]		
	$K_{I}$ [MPa·mm^1/2^]		$K_{I}^{\max}$ [MPa·mm^1/2^]	ϕ	d	$K_{I}^{\max}$ [MPa·mm^1/2^]	ϕ	d
	ϕ=0°	ϕ=90°						
1	609.75	767.11	781.18	90°	1.80%	763.86	90°	0.43%
2	864.44	1047.08	1059.85	90°	1.20%	1032.52	90°	1.41%
3	1174.84	1316.22	1375.78	90°	4.33%	1324.32	90°	0.61%
4	1505.27	1503.32	1819.53	90°	17.38%	1514.12	0°	0.58%
5	1632.57	1562.12	2049.56	90°	23.78%	1631.14	0°	0.09%
6	1737.65	1608.92	2309.72	90°	30.34%	1726.55	0°	0.64%
7	1762.95	1621.54	2392.61	90°	32.23%	1750.65	0°	0.70%
8	1815.73	1648.81	2591.64	90°	36.38%	1799.13	0°	0.92%
9	1828.87	1655.93	2650.30	90°	37.52%	1817.99	0°	0.60%
10	1930.93	1670.63	3215.65	90°	48.05%	1896.17	0°	1.83%
11	1944.21	1680.66	3332.81	90°	49.57%	1915.37	0°	1.51%
12	1951.63	1705.72	3347.023	90°	49.04%	1922.46	0°	1.52%
